# Meteorological Observations

**Published:** 1859-09

**Authors:** J. G. Westmoreland

**Affiliations:** Smithsonian, Institution, Aug., 1859, at Atlanta, Ga.


					﻿22	,	61	50 X X X TO	XXX	TOx XXX
s	.
■|5 ■ s	I
■5	>£ <N	X x 32 X	X	m	XX US XX
Is
*S '	_________________________________________
S ~ I	---'
<3 C5 |	u
s yo	.. pq m . fe ....... . rq . .	. . . cj a ri r
££__________ ' »
I--------------------------------------- 1
■S*	d	3
i|	*	"'^'"222SS222l0l-’'- = c = 2ooooo = cCoL,oL-1 3
'	°	r
r f ____________	_	Z
A J	s	         >
co	;	p «	g
8	-	■°”’°'°“’'022S2223'»2’»»°» = o = oO = ,^^„«5 5
•g-s	001	’2
"§>	2	'	a
1|	<	2^22222222°^OOOOOOeoOOOOOO« |
gj •__________ ______ s
2 £ s	s § ass§ g	gf-----s— “
fk O	<	rH	CO	_	«
CO	P5	I,
CO ------------------ ;j
si I
g C | i W	---------------------------------_-------■ o
2	i~roaoco;x>2iSL2itf22r^2oortooaoooSgS22 — ° °-i -r o 2
•ip • JS „	1 i co 1 co co °o <» co co co o r- co oo co co t- t- oo s
J?	;	a	_2!—______________________________________  	<
ss ^b	a	g	  ——■——   5
•.§ _g	h	«j	'oco^^i^t2o2®oo22tir222£J£}22>SG:>c’<N':,*,>1cq<=><:<’c':’	&
~	, L1I~ l~ l~ l~ O O I- O O O I- O O
S ~SS22Sggg2Ssligli¥g^ll^gg222gs’sf
S	I	*	i-SSSsl^s'Iill^I^iissigisssfs^
>■	§	.5.
1	”	a	If555S?®®®«§s^®ss®sssssis22ggg¥
■§	_ H~cocoTO«gggg-g®g^<go®ooooooooooooo
				

